# Chemogenetic inhibition of the ventral hippocampus but not its direct projection to the prelimbic cortex attenuates context-specific operant responding

**DOI:** 10.3389/fnbeh.2024.1310478

**Published:** 2024-02-07

**Authors:** Callum M. P. Thomas, Mark E. Bouton, John T. Green

**Affiliations:** ^1^Department of Psychological Science, University of Vermont, Burlington, VT, United States; ^2^Neuroscience Graduate Program, University of Vermont, Burlington, VT, United States

**Keywords:** ventral hippocampus, prelimbic, context, operant, Pavlovian, DREADD

## Abstract

Previous work has demonstrated the importance of the prelimbic cortex (PL) in contextual control of operant behavior. However, the associated neural circuitry responsible for providing contextual information to the PL is not well understood. In Pavlovian fear conditioning the ventral hippocampus (vH) and its projection to the PL have been shown to be important in supporting the effects of context on learning. The present experiments used chemogenetic inhibition of the direct vH-PL projection or the vH to determine involvement in expression of context-specific operant behavior. Rats were injected with an inhibitory DREADD (hM4D_i_) or mCherry-only into the vH, and subsequently trained to perform a lever press response for a food pellet in a distinct context. The DREADD ligand clozapine-n-oxide (CNO) was then delivered directly into the PL (experiment 1) and then systemically (experiment 2) prior to tests of the response in the training context as well as an equally familiar but untrained context. vH (systemic CNO) but not vH-PL (intra-PL CNO) inhibition was found to attenuate operant responding in its acquisition context. A third experiment, using the same rats, showed that chemogenetic inhibition of vH also reduced Pavlovian contextual fear. The present results suggest that multisynapatic connections between the vH and PL may be responsible for integration of contextual information with operant behavior.

## 1 Introduction

The prelimbic cortex (PL) is well established as a critical region in mediating the effects of context on operant behavior. Previous work has shown that PL inactivation can attenuate renewal of drug seeking behaviors (Fuchs et al., 2007; Willcocks and McNally, 2013; Palombo et al., 2017), and interrupt the contextual control of response conflict (Marquis et al., 2007). Similarly, we have consistently found that PL inactivation attenuates operant responding when tested in the context in which it was trained (i.e., its acquisition context), but has no effect when responding is tested in a context where it was never trained (Eddy et al., 2016; Trask et al., 2017; Thomas et al., 2020). Additionally, we have shown that the PL is involved in the ability of many different types of contexts (background stimuli), including satiety and stress interoceptive states, and previous behaviors, to affect performance of operant responses (Thomas et al., 2020, 2023a,b). The diverse nature of these types of contexts suggests that the PL may function as a hub in which contextual information is integrated with behavioral output.

Previous work has shown that the ventral hippocampus (vH) is also involved in contextual effects on behavior. Most of this work has been in Pavlovian conditioning paradigms (for review see Bouton et al., 2021). vH connections with the PL have been found to be involved in acquisition, expression, and renewal of conditioned context fear (Vasquez et al., 2019; Hallock et al., 2020; Santos et al., 2020; Twining et al., 2020). For example, Twining et al. (2020) optogenetically silenced vH terminals in the PL during trace fear conditioning and at test found reduced context fear expression but no effect on cued fear expression. Similarly, ABA renewal of appetitive Pavlovian responding has been found to correlate with activation (Fos induction) of ventral hippocampal neurons that project to the PL (Anderson and Petrovich, 2018).

Collectively, past results suggest that the vH may serve as a source of contextual information to the PL. While this has been primarily shown in Pavlovian conditioning, a few studies have demonstrated a role of the vH and its projections to nucleus accumbens shell and infralimbic cortex in renewal of instrumental drug seeking behaviors (Atkins et al., 2008; Lasseter et al., 2010; Bossert and Stern, 2014; Marchant et al., 2016; Wang et al., 2018). The present experiment was designed to test the hypothesis that in addition to its identified role in context-mediated Pavlovian conditioning, the vH-PL pathway is involved in the expression of context-mediated operant responding. To that end, we expressed an inhibitory DREADD [CaMKIIa-hM4D(Gi)] in the ventral hippocampus. Activation of inhibitory DREADDs induces hyperpolarization of the cell bodies of expressing neurons and also induces synaptic silencing of axon terminals. We made use of these two mechanisms to selectively inhibit the vH-PL pathway (via intra-PL infusions of CNO) (Experiment 1) or the entire vH (via I.P. CNO injection) (Experiment 2). In each experiment, a lever press response was tested in both the context where it had been trained (context A) and an equally familiar but untrained context (context B). Given our hypothesis, we expected both treatments to selectively reduce responding in the acquisition context. We additionally tested the effects of vH inactivation on Pavlovian context fear (Experiment 3), as a positive control for the effectiveness of vH inactivation, since vH inactivation has been shown in many studies to attenuate Pavlovian context fear (e.g., Esclassan et al., 2009; Gilmartin et al., 2012).

## 2 Materials and methods

### 2.1 Subjects

The subjects were 32 male Wistar rats purchased from Charles River Laboratories in two cohorts (*n* = 16). Rats were between 59 and 63 days old at delivery and were housed in a room maintained on a 12:12 h light:dark cycle. All experimental procedures took place during the light period of the cycle.

### 2.2 Surgery

Following a 5-day acclimation period housed in pairs, rats were anesthetized with isoflurane and stereotaxic surgery was performed to bilaterally implant guide cannulae (22 gauge, Plastics One) in the PL region of the mPFC. Guide cannulae were lowered into the brain, using a 12-degree angle in the mediolateral plane, to the target coordinates (AP: +3.0 from bregma, ML: +/− 0.75, DV: −3.1 mm). The inhibitory DREADD viral construct pAAV-CaMKIIa-hM4D(Gi)-mCherry (gift from Bryan Roth; Addgene viral prep # 50477-AAV8; http://n2t.net/addgene:50477; RRID:Addgene_50,477) or the control viral construct pAAV-CaMKIIa-mCherry (gift from Karl Deisseroth; Addgene viral prep # 114469-AAV8; http://n2t.net/addgene:114469; RRID:Addgene_114,469) was infused bilaterally into the ventral hippocampus with a Hamilton syringe (AP: −6.0 from bregma, ML: +/− 5.9, DV: −7.0 mm). Prior to each infusion, the needle was lowered into place and allowed to settle for 1 min. Then 1.0 μL of virus was infused at a rate of 0.1 μL/min and the needle remained in place for 5 additional minutes to allow for diffusion before being slowly retracted. The hole drilled into the skull for each infusion was then filled with sterile bone wax (Medline).

During surgery, topical Lidocaine-Prilocaine cream (2.5%/2.5%; approx 0.25 mL) was administered as a local anesthetic, lactated Ringer’s (1 mL; s.c.) was administered for hydration, and carprofen (5.0 mg/kg; s.c.) was administered for analgesia. A second dose of carprofen was administered 24 h postoperatively. Rats were subsequently single-housed and maintained on *ad libitum* chow access for 6 weeks following surgery before being food deprived and maintained at 90% of their baseline weight for the remainder of the experiment.

### 2.3 Apparatus

Experimental procedures were conducted in four sets of four conditioning chambers (Med Associates model ENV-008-VP) housed in individual sound attenuating chambers. Two sets of four chambers were used for operant conditioning (Operant chambers) and were housed in two separate rooms of a laboratory. The second two sets of chambers were used for context fear conditioning (Pavlovian chambers) and were housed in a single room in a separate laboratory.

Each operant chamber, measuring 30.5 × 24.1 × 21.0 cm (l X W X h), had a recessed 5.1 × 5.1 cm food cup (magazine) centered in the front wall. A retractable lever (Med Associates model ENV-112CM) was positioned to the left of the food cup and extended 1.9 cm into the chamber when inserted. The sound attenuation chamber was lit by one 7.5 W incandescent bulb mounted approximately 34.9 cm from the grid floor at the front of the chamber. Ventilation fans provided background noise of 65 dBA (measured inside the conditioning chamber).

One set of operant chambers had a staggered height steel rod flooring (0.48 cm diameter rods with 0.5 cm height difference between neighboring rods), clear acrylic sidewalls and ceiling, brushed aluminum rear and front walls, and included a dish containing 5 mL of lemon-scented Pine-Sol (Clorox) outside the operant chamber. The second set of boxes was identical to the lemon-scented boxes except one side wall and the acrylic ceiling had black diagonal strips (3.8 cm wide and 3.8 cm apart), the rod flooring was all mounted in the same plane (1.6 cm apart), and a scent was provided by Pine-Sol (Clorox). All boxes delivered the same reinforcer, a 45 mg sucrose pellet (5-TUT:1811251; TestDiet, Richmond, IN).

In comparison to the operant chambers, the Pavlovian chambers measured 30.5 × 24.1 × 29.0 cm (l X W X h) and were lit by a house light (Med Associates, ENV-215 M) centered on the back wall 24 cm above the floor. One set of boxes was scented with 20% anise extract diluted in water (McCormick & Co) and the other with 10% coconut extract diluted in water (McCormick & Co). Anise-scented boxes’ flooring consisted of alternating 0.4 cm and 0.9 cm diameter stainless steel rods (placed 1.6 cm apart center-to-center) and ventilation fans provided 68 dB background noise. Side walls were also decorated with a horizontal black bar and a geometric insert was attached at a 45-degree angle to the rear and side wall. In comparison, the coconut-scented boxes floor consisted of 0.4 cm stainless steel rods arranged in an arch. The ceiling and one side wall were decorated with a blue polka dot pattern and exhaust fans provided background noise (68 dB).

### 2.4 Experimental procedures

Experimental procedures began 7 weeks following viral vector infusion and were completed within 2 weeks.

#### 2.4.1 Drug

Prior to each test, rats received either intracranial (IC) (Experiment 1) or intraperitoneal (IP) (Experiments 2 and 3) deliveries of CNO. IC delivery consisted of 0.5 μL bilateral infusions of CNO (1.0 mM) infused at a rate of 0.25 μL/min. Internal cannulae tips protruded 1 mm below the tip of guide cannulae and were left in place for 2 min before being removed and replaced with dummy cannulae. Rats were then transported to operant chambers after a delay of 5 to 15 min. IP delivery consisted of a single IP injection of CNO (3 mg/kg) first dissolved in dimethyl sulfoxide (DMSO; 1% of final volume) and then in sterile phosphate buffered saline (PBS) to reach a final concentration of (2 mg/mL). IP injections were completed in the colony room and occurred 30 min before test sessions.

#### 2.4.2 Lever-press acquisition

Throughout behavioral procedures, rats received sessions in their acquisition context (context A) and in an untrained context (context B). The actual boxes providing the two contexts were counterbalanced. The order of sessions was also counterbalanced and alternated daily. On the first day of experimental procedures, rats received a magazine training session in both their context A and their context B. Magazine training sessions consisted of a 2-min delay followed by free delivery of 30 sucrose pellets to the magazine according to a RT60 (random time 60 s) schedule. On the following 6 days, rats received lever-press acquisition sessions which consisted of the same 2-min delay followed by insertion of the lever. Lever-press responses were reinforced by delivery of sucrose pellets according to a variable interval 30 s schedule (VI30) such that on average a pellet became available every 30 s. On each day rats also received an exposure session of equal duration to their context B, where the lever was never inserted.

#### 2.4.3 Experiment 1 - vH➔ PL inhibition operant tests

On the day after the last session of acquisition, rats received a test of the lever press response in their context A and in their context B. Tests were identical to acquisition sessions except that they were 10-min in duration and did not include delivery of sucrose pellets (i.e., tests occurred in extinction). A minimum of 5 min prior to their first test, all rats received intra-PL infusions of CNO (as detailed above). Between the two tests, rats were returned briefly to their home cage such that tests were separated by approximately 10 min.

#### 2.4.4 Experiment 2 - vH inhibition operant tests

Following the vH-PL inhibition operant tests, rats received two additional days of acquisition sessions to allow for recovery of response rates and to allow for intracranial CNO clearance. Subsequently, 30 min after intraperitoneal CNO delivery (based on their current weight) rats again received tests of the lever press response in each context. Test sessions were otherwise identical to the tests which occurred following intracranial CNO delivery. Following their final test, rats were returned to their home cage and were given *ad libitum* access to chow.

#### 2.4.5 Experiment 3 - vH inhibition Pavlovian context fear test

Three days later, rats were placed in their novel chamber and after a three-minute delay 3 foot shocks (1 mA, 2 s each) were delivered with a 60-s inter-shock interval. One minute after the third shock, rats were returned to their home cage. On the following day all rats received an IP CNO injection and after a 30-min delay were placed back in the same chamber for 20 min.

### 2.5 Video analysis

Automated scoring of freezing was conducted using the following method: video streams were acquired in near-infrared (720P resolution, 29.97 frames per second) by Anpviz IPCameras (model IPC-B850W) mounted in each chamber. Streams were delivered over a dedicated ethernet network and captured by a computer running ffmpeg. Recordings were subsequently scored by first computing the absolute difference in pixel intensity at every pixel on each pair of subsequent frames. A per-frame activity measure was produced by averaging this difference over all pixels. Inspection of the distribution of (log10-transformed) activity scores revealed a clear bimodal distribution of activity, with the mode of the lowest scores reflecting video noise and mode of the higher scores reflecting rat movement. These distributions varied almost solely by chamber/camera. Presumptive freezing was therefore defined as occurring, on a per-chamber basis, when the activity score fell below the value visually marking the beginning of the rat-movement related portion of the distribution. Activity scores were then averaged in 1 s bins, and only 1 s bins that fell below the threshold were defined as representing freezing [approximating procedures used by the Fanselow laboratory, e.g., Fanselow et al. (2019)]. This method for algorithmically scoring freezing was previously found to correlate well with freezing scored by trained human observers in a separate experimental preparation, with all r-values exceeding 0.80. Videos were further screened to exclude immobility due to sleeping from the freezing measure.

### 2.6 Histology

**Figure 1:**
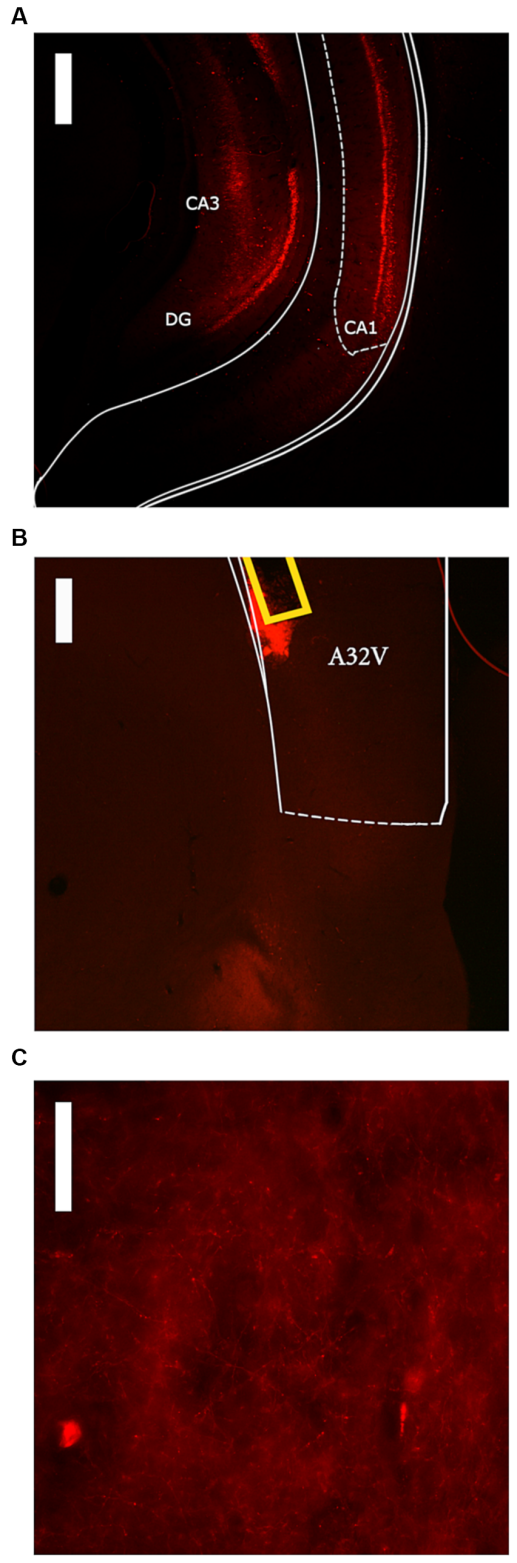
Representative images of DREADD expression within the High DREADD expression group. **(A)** ventral hippocampus expression (score = 3) at 2.5x magnification with 500 μM scale bar. **(B)** prelimbic cortex (A32V) expression at 4x magnification with 300 μM scale bar. Cannula tract shown in yellow. **(C)** prelimbic cortex expression at 40x magnification with 50 μM scale bar.

Following completion of the experiment, all rats received a lethal dose of sodium pentobarbital (150 mg/kg, i.p.) before being transcardially perfused with 0.9% saline followed by 10% buffered formalin. Brains were removed and stored in 10% buffered formalin for 1 h and then stored in a 30% sucrose/PBS solution for cryoprotection. Brains were then sectioned at 60 μm on a cryostat and mounted onto chromium aluminum subbed slides. Once slices were stable, slides were coverslipped with DAPI Fluoromount-G (SouthernBiotech) and edges were sealed with clear nail polish. Cannula placements and viral expression were then confirmed using a compound fluorescent microscope (Zeiss Axioskop I). DREADD-mCherry expression in the ventral hippocampus was scored according to a scale of 1–4 by an observer blind to behavioral data. A score of 1 indicated visible expression in the ventral hippocampus but with limited spread (1 mm or less), whereas a score of 4 indicated substantial expression and spread throughout the ventral hippocampus. A representative image of a score of 3 is shown in Figure 1A. The DREADD group was additionally divided into high and low expression groups using criteria described below.

## 3 Results

Following analysis of viral expression in the vH, one rat was excluded from subsequent analyses because viral expression was exclusively outside the vH. Two mCherry control rats were excluded from analysis because they did not reach the end of the experiment due to health issues (final *n* = 14). Three mCherry control rats were found to have cannula placements posterior to the boundary of Brodmann’s area 32; however, their response rates during IC operant tests did not differ from the remaining control rats so they were included in analyses.

**Figure 2:**
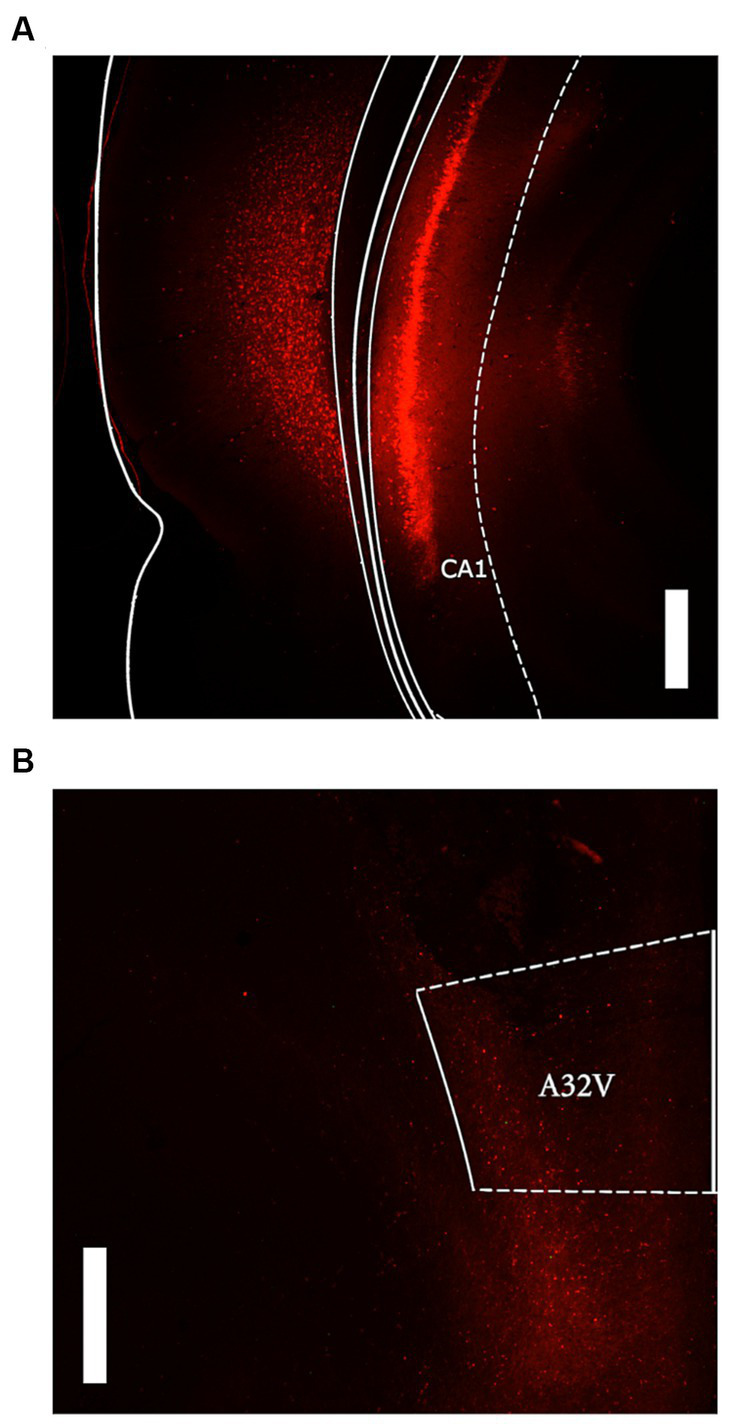
Representative images of mCherry control expression. **(A)** Ventral hippocampus expression at 2.5x magnification with 500 μM scale bar. **(B)** prelimbic cortex (A32V) expression at 2.5x magnification with 500 μM scale bar.

**Figure 3:**
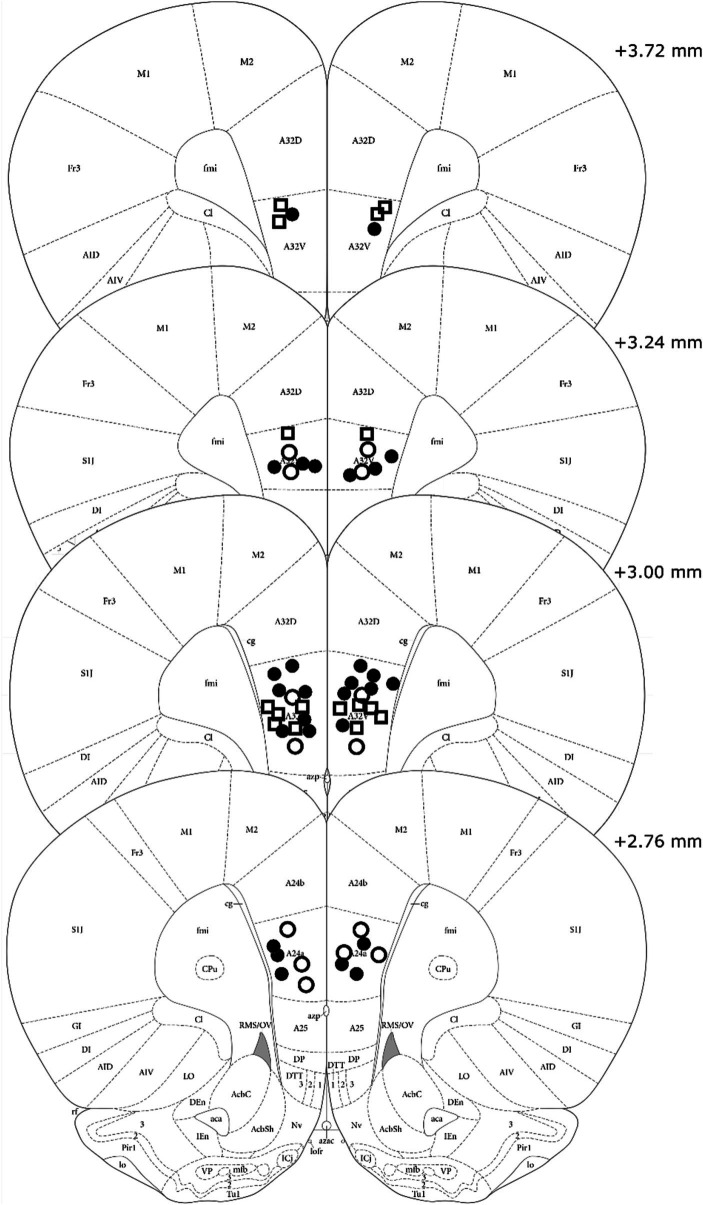
Cannula tip placements of all rats included in analysis. Treatment group is indicated by shape and fill (High DREADD – open square, Low DREADD – open circle, mCherry control – closed circle). Atlas panels adapted from Paxinos and Watson (2014).

Visual analysis of DREADD-mCherry expression in the PL indicated that it strongly depended on expression in the vH. At 4x and 10x magnification, DREADD-mCherry expression in the PL was only visible in hemispheres with ipsilateral vH scores of 3 or 4. In comparison, at 40x magnification axonal expression in the PL was dense in hemispheres with strong ipsilateral vH expression (scores 3 and 4) but sparse in hemispheres with weak ipsilateral vH expression (scores 1 and 2). Based on this difference in expression in PL terminals, the DREADD expressing group was split into high and low expression groups (*n* = 8 and *n* = 7, respectively). Examples of PL DREADD expression ipsilateral to vH which received a score of 3 are shown in Figures 1B,C. To ensure analytical rigor, we analyzed test data from each experiment using the original DREADD expression group and separately with these high and low expression groups (see below). The expression of mCherry in control animals did not significantly vary across subjects and was generally more intense and spread farther from the injection site than DREADD-mCherry expression (see Figures 2A,B). While intensity of fluorescence differed significantly between DREADD and mCherry control animals, the pattern of expression throughout the vH and PL did not. Three rats with low DREADD expression in the PL were also found to have cannula placements posterior to the boundary of Brodmann’s area 32. Considering their placement in the low DREADD expression group and that their response rates did not differ from other low DREADD expression animals during IC operant tests they were included in analyses. Distribution of cannula placements are depicted in Figure 3.

### 3.1 Experiment 1 - vH➔ PL inhibition operant tests

**Figure 4:**
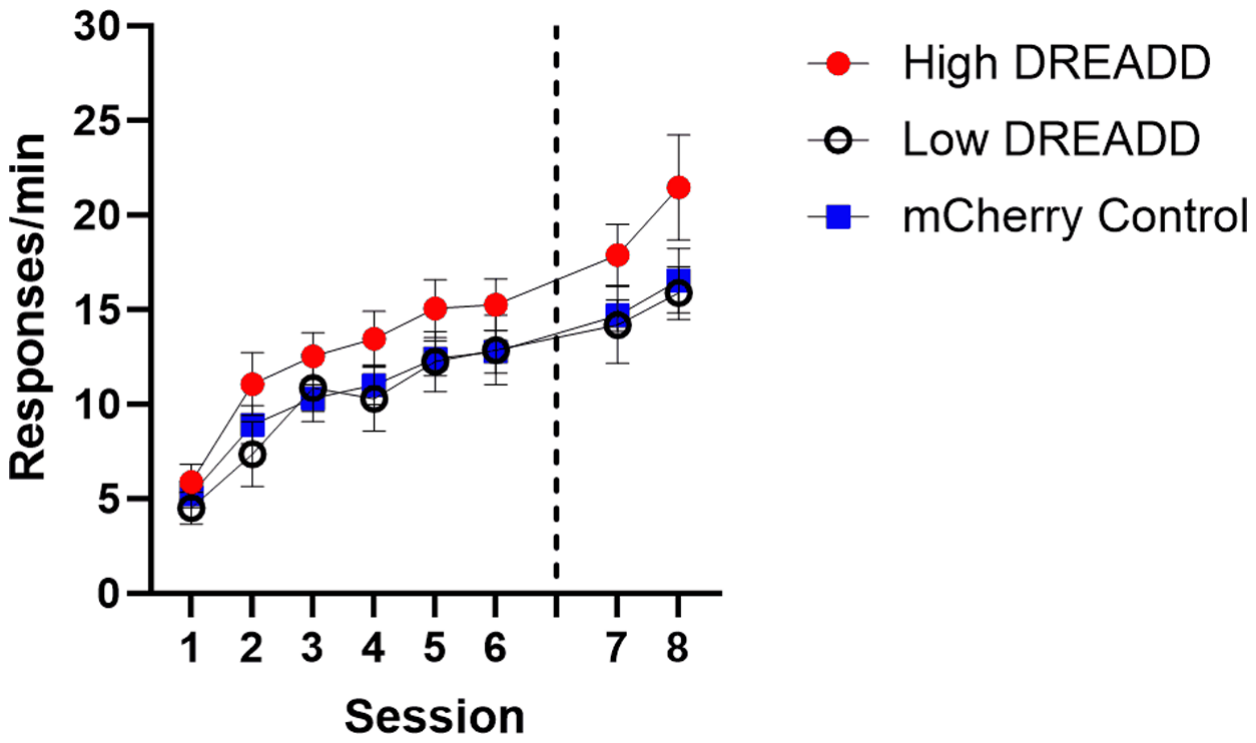
Operant Response Acquisition. Response rates (per min) for each group during the initial six and two additional sessions of training. Dotted line indicates when vH-PL inhibition operant tests occurred. Error bars indicate standard error of the mean.

Acquisition data were analyzed using a 6 (Session) x 3 (Group: High, Low, Control) repeated measures ANOVA. A significant main effect of Session indicated that all groups acquired the lever press response, *F* (5, 130) = 46.79, MSE = 5.53, *p* < 0.001. No other main effects or interactions reached significance, largest *F* = 1.53. However, response rates in the High DREADD expression group were generally higher than the other two groups so test data were converted to a proportion baseline score, using response rates on day 6 (Test response rate/Acquisition day 6 response rate) (see Figure 4).

**Figure 5:**
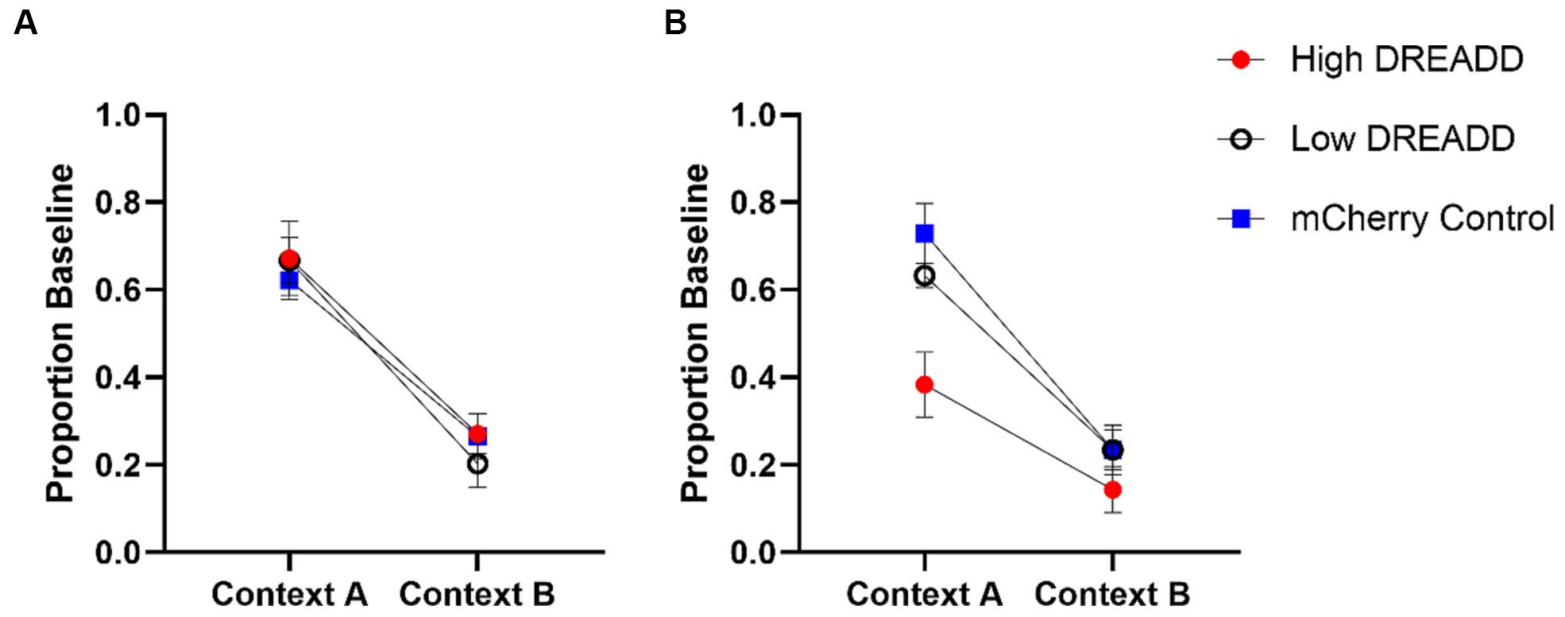
Operant Test Results. vH-PL inhibition **(A)** and vH inhibition **(B)** operant test responding as a proportion of baseline (response rate during the acquisition session occurring on the day prior to each test). Error bars indicate standard error of the mean.

Test data were initially analyzed using a 2 (Context: A, B) x 2 (Group: DREADD, Control) repeated measures ANOVA. A significant main effect of Context indicated that response rates were greater in context A than in context B in all groups, *F* (1, 27) = 75.76, MSE = 0.03, *p* < 0.001. No other main effects or interactions approached significance, largest *F* = 0.68. Test data were then analyzed using a 2 (Context: A, B) x 3 (Group: High, Low, Control) repeated measures ANOVA (see Figure 5A). A significant main effect of Context was again observed, *F* (1, 26) = 72.21, MSE = 0.03, *p* < 0.001, but no other main effects or interactions reached significance, largest *F* = 1.03 (see Figure 5A). Given the *a priori* hypothesis that DREADD inhibition of the vH-PL pathway would attenuate context-specific operant responding, we additionally conducted a post-hoc analysis using Tukey’s multiple comparisons test, which confirmed that no group differed from any other in either context (all *p’s* > 0.72).

### 3.2 Experiment 2 - vH inhibition operant tests

Acquisition data from the 2 days between Experiment 1 and the Experiment 2 operant tests were analyzed using a 2 (Session) x 3 (Group: High, Low, Control) repeated measures ANOVA (see Figure 4). A significant main effect of Session indicated that response rates increased in all groups, *F* (1, 26) = 6.04, MSE = 12.40, *p* = 0.021. No other main effects or interactions were significant, largest *F* = 2.41. However, a (nonsignificant) mean difference between Group High and Groups Low and Control was still present, so proportion baseline scores were once again calculated for test results using the previous day’s response rates.

Experiment 2 test data were initially analyzed using a 2 (Context: A, B) x 2 (Group: DREADD, Control) repeated measures ANOVA. A significant main effect of Context indicated that response rates were again greater in context A than in context B across all groups, *F* (1, 27) = 76.11, MSE = 0.03, *p* < 0.01. A significant effect of Group was also revealed, *F* (1, 27) = 5.80, MSE = 0.47, *p* = 0.023. A Group by Context interaction approached but did not quite attain statistical significance, *F* (1, 27) = 3.76, MSE = 0.03, *p* = 0.06. Experiment 2 test data were then analyzed using a 2 (Context: A, B) x 3 (Group: High, Low, Control) repeated measures ANOVA (see Figure 5B). We again observed a significant main effect of Context, *F* (1, 26) = 62.65, MSE = 1.89, *p* < 0.01, and a significant effect of Group, *F* (2, 26) = 6.03, MSE = 0.25, *p* < 0.001. Post-hoc analysis using Tukey’s multiple comparisons test confirmed that Group High’s response rates were on average significantly lower than Group Control (*p* = 0.006) but not Group Low (*p* = 0.075). Again a Group by Context interaction approached but did not attain statistical significance, *F* (2, 26) = 2.74, MSE = 0.03, *p* = 0.08. However, given our *a priori* hypotheses, we additionally conducted a post-hoc comparison of each group’s response rates in each context using Tukey’s multiple comparisons test. These tests revealed that response rates in Group High were significantly lower than Groups Low (*p* = 0.036) and Control (*p* < 0.001) in context A, whereas no groups differed in context B (all *p’s* > 0.53).

### 3.3 Experiment 3 - vH inhibition Pavlovian context fear test

To ensure previous treatments did not affect acquisition of context fear, freezing throughout the acquisition session was analyzed using a 6 (Minute) x 3 (Group: High, Low, Control) repeated measures ANOVA. A main effect of Minute indicated that on average freezing increased across minutes in the chamber, *F* (5, 130) = 57.04, MSE = 79.49, *p* < 0.001. No other main effects or interactions approached significance, largest *F* = 1.07.

**Figure 6:**
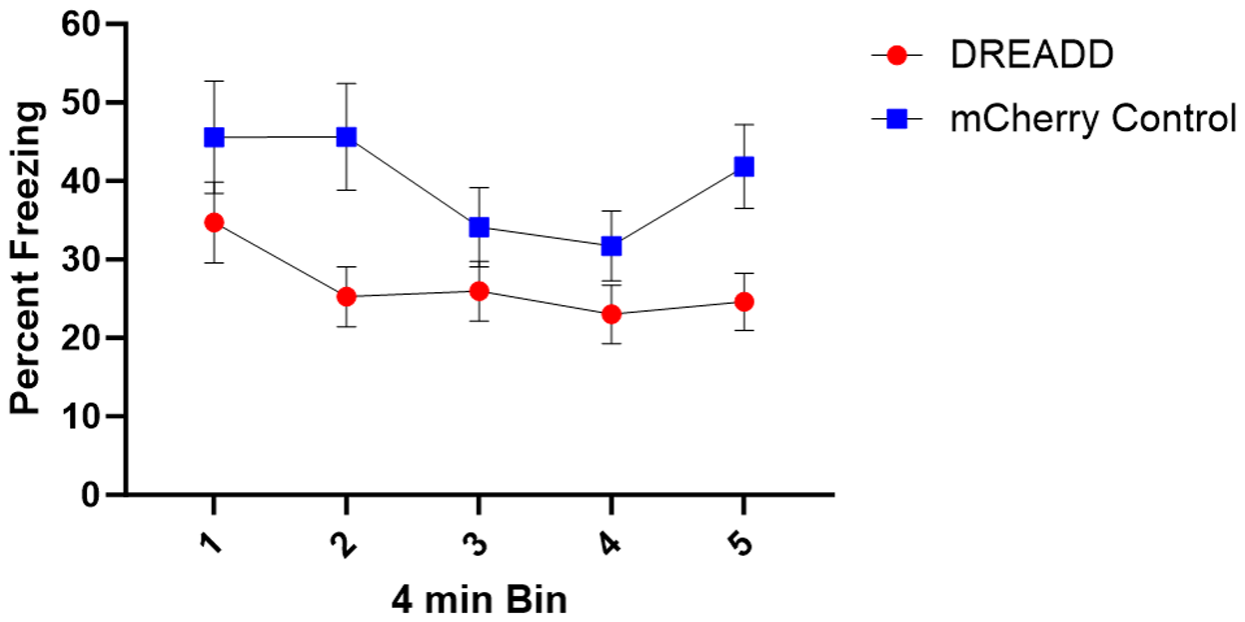
Context Fear Test Results. Mean percent time freezing during 4-min bins of the context fear test. Error bars indicate standard error of the mean.

Context fear test data were divided into five 4-min bins and then analyzed with a 5 (Bin) x 2 (Group: DREADD, Control) repeated measures ANOVA (see Figure 6). This analysis revealed a significant main effect of Bin, indicating that freezing decreased across bins *F* (4, 108) = 7.32, MSE = 96.09, *p* < 0.001. Additionally, a main effect of Group indicated that the DREADD group exhibited less freezing than the Control group, *F* (1, 27) = 4.39, MSE = 1410.35, *p* = 0.046. No other main effects or interactions were significant, largest *F =* 2.23. Subsequent analysis using a 5 (Bin) x 3 (Group: High, Low, Control) repeated measures ANOVA similarly produced a significant main effect of bin, *F* (4, 104) = 5.80, MSE = 94.85, *p* < 0.001. In contrast with the initial analysis, however, no main effect of Group was observed, *F* (2, 26) = 2.45, MSE = 1432.24, *p* = 0.11. This difference may simply be the result of a reduction in statistical power, however, because follow-up analysis of the two DREADD expression groups, using a 5 (Bin) x 2 (Group: High, Low) repeated measures ANOVA did not reveal any significant effects involving Group, largest *F* = 1.58, suggesting that in this test degree of DREADD expression in PL terminals did not predict a greater reduction in freezing.

## 4 Discussion

The present experiments utilized chemogenetic inhibition (hM4Di-DREADD) to investigate the involvement of the vH and its projection to the PL in the performance of an operant behavior in both its acquisition context and an equally familiar but untrained context. To examine vH projections to PL, we bilaterally infused a hM4Di-DREADD virus into vH and bilaterally cannulated the PL. In Experiment 1, intracranial infusion of CNO into the PL was used to produce vH-PL pathway-specific synaptic silencing. In Experiment 2, systemic (intraperitoneal) delivery of CNO was used to inhibit the vH and all its projections. Given the previously identified role of the PL in acquisition context-specific operant responding, we hypothesized that both vH-PL pathway-specific inhibition and general vH inhibition would attenuate responding in the acquisition context. In contrast with this prediction, however, we found that only vH inhibition attenuated operant responding in the acquisition context.

The absence of an effect with vH-PL pathway-specific inhibition has several possible explanations. First, it is possible that the vH-PL projection is not involved in the expression of context-specific operant responding. The vH also projects to a number of other PL projecting brain regions (including the mediodorsal thalamus, infralimbic cortex, and basolateral amygdala). Considering the attenuation of operant responding we observed following inhibition of the vH, it seems plausible that a multisynaptic connection between the vH and PL might be involved in conveying contextual information to the PL. Additionally, the absence of a vH-to-PL inactivation effect in the current experiment does not necessarily indicate the absence of a role in this projection in contextual control of behavior; additional multisynaptic connections could serve as redundant connections between the vH and PL which individually only become critical for behavior under specific circumstances (e.g., spatial navigation within a specific context vs. general representation of the context). In such a case, our behavioral paradigm might have allowed for alternative multisynaptic connections to transmit contextual information to the PL in the absence of activity along the direct pathway, whereas inhibition of the entire vH prevents communication entirely. A similar argument was proposed by Ito et al. (2015) to explain why lesions of the nucleus reuniens, which relays inputs from the mPFC to the hippocampus, could eliminate trajectory coding in hippocampal CA1 place cells during a T-maze-based alternation task without affecting behavioral performance. Instead, CA1 trajectory coding might only become critical to performance in more complex behavioral tasks. As such, it may still be worthwhile to investigate whether activity in vH-to-PL neurons correlates with features of context-based operant tasks.

Previous work has shown the involvement of vH-PL projections in Pavlovian context fear expression and ABA renewal (Vasquez et al., 2019; Hallock et al., 2020). It is possible that the direct vH-PL pathway might be preferentially involved in Pavlovian conditioning but not operant conditioning or in aversive but not appetitive conditioning. However, it is important to note that these previous studies used a somewhat different method from ours to manipulate the vH-PL pathway. For example, Hallock et al. (2020) infused a retrograde Cre-expressing virus into the PL and a Cre-dependent DREADD virus into the vH. An important distinction between this method and the one used here is that the systemic injection of CNO used in this dual-virus approach may affect all collateral projections of any vH neuron that projects to the PL in addition to direct projections. Interestingly, previous work in mice has suggested that 62% of vCA1 neurons which project to the mPFC also project to at least one other region (Gergues et al., 2020; see supplementals). This suggests that while the number of direct projections to the PL inhibited by each method might be the same, the dual virus approach may also inhibit many non-PL projecting collaterals.

Another possible explanation for the null effect we observed when manipulating the vH-PL pathway is that there was not enough DREADDs expression at vH terminals in the PL to sufficiently attenuate synaptic activity. In an attempt to determine if this was the case, we evaluated whether the degree of DREADD expression at vH terminals in the PL was related to the effect of treatment on acquisition context responding. Interestingly, the high DREADD expression group did not differ behaviorally from either the low DREADD expression group or controls during the vH-PL tests, despite the fact that we observed significant DREADD expression within the PL and in close proximity to cannulae tips in these animals. In line with previous work, we also observed expression preferentially within PL layer 5 and throughout the ventral aspects of the medial prefrontal cortex (mPFC) (e.g., infralimbic cortex) (Liu and Carter., 2018). Notably, the level of DREADDs expression in the PL did modulate the effect of attenuation of vH, through peripheral CNO injection on context-dependent behavior, though it remains unclear if this effect was dependent on expression of the DREADD within the mPFC as this expression covaried with intensity and spread of expression within the vH.

In Experiment 3, we found that inhibition of the vH (by IP CNO delivery) reduced context-fear expression, replicating past findings (e.g., Hobin et al., 2006; Sierra-Mercado et al., 2011; Zhu et al., 2014; Park et al., 2020). The attenuation of context freezing we observed also served as a positive behavioral control for our DREADD manipulation and as a point of comparison with another context-dependent behavioral phenomenon. Additionally, the finding that peripheral CNO during this test increased activity (i.e., decreased freezing) suggests that the reduction of lever-pressing in the operant tests was not likely a result of motor effects.

One important limitation of the design of the present experiments is that we did not counterbalance the order of the vH➔PL operant, vH operant, and vH Pavlovian context fear tests. (All animals received the tests in that order.) However, previous work has suggested that repeated activation of hM4Di DREADDs does not produce appreciable receptor downregulation or behavioral effects unrelated to its temporally restricted effects on neuron excitability (Roth, 2016). Additionally, vH-PL tests only involved delivery of CNO to the PL, limiting any possible effects to those specific vH projections during the IP operant tests. Nonetheless, the order of testing should be considered when interpreting the results. Another important point of note is that experiments 2 and 3 both used systemic delivery of CNO, which can have off-target effects, including increased feeding in rodents (Mac Laren et al., 2016; Gomez et al., 2017; Abela et al., 2020). In the present experiments, however, both DREADDs and non-DREADDs control groups received CNO. Finally, the present experiments used only male rats. Future research would benefit from inclusion of both sexes.

Collectively, the present results support the idea that the vH is involved in the expression of context-dependent operant and Pavlovian fear responding, but that the vH-PL pathway is not necessary for context-dependent operant responding. Future work might investigate the possibility of multisynaptic connections between the vH and the PL in supporting context-dependent operant behavior.

## Data availability statement

The raw data supporting the conclusions of this article will be made available by the authors, without undue reservation.

## Ethics statement

The animal study was approved by University of Vermont Institutional Animal Care and Use Committee. The study was conducted in accordance with the local legislation and institutional requirements.

## Author contributions

CT: Conceptualization, Data curation, Formal analysis, Investigation, Methodology, Project administration, Software, Validation, Visualization, Writing – original draft, Writing – review & editing. MB: Conceptualization, Funding acquisition, Methodology, Resources, Writing – review & editing. JG: Conceptualization, Funding acquisition, Methodology, Resources, Supervision, Writing – review & editing.
